# Scale-up fermentation of *Escherichia coli* for the production of recombinant endoglucanase from *Clostridium thermocellum*

**DOI:** 10.1038/s41598-021-86000-z

**Published:** 2021-03-30

**Authors:** Iram Shahzadi, Maryam A. Al-Ghamdi, Muhammad Shahid Nadeem, Muhammad Sajjad, Asif Ali, Jalaluddin Azam Khan, Imran Kazmi

**Affiliations:** 1 Institute of Molecular Biology and Biotechnology, University of Lahore, Defence Road Campus, Lahore, 54590 Pakistan; 2 Department of Biochemistry, Faculty of Science, King Abdulaziz University, Jeddah, 21589 Saudi Arabia; 3 School of Biological Sciences, University of the Punjab, Lahore, 54590 Pakistan

**Keywords:** Biotechnology, Microbiology

## Abstract

Endoglucanase (EC 3.2.1.4) catalysing the hydrolysis of β-1.4-glycosidic linkage of cellulose molecules is an enzyme of tremendous industrial importance. The present study describes a response surface methodology based predicted model to deduce a set of fermentation conditions for optimum growth and activity of recombinant endoglucanase in *E. coli* BL21 (DE3). Numerous significant parameters including fermentation media composition, temperature (Celsius), pH and agitation rate (rpm) were analysed systemically by employing central composite design. This effort reports highly efficient recombinant endoglucanase overproduction (6.9 gl^−1^ of biomass) with 30% expression by *E. coli* in modified M9NG media incubated at 37 °C and pH 7 agitated at 200 rpm. Addition of 3 mM glucose and 24 mM glycerol in the M9NG media has shown positive effect on the enzyme yield and activity. The CMCase activity experimentally estimated was found to be 1185 U/mg with the optimized parameters. The outcomes of both the responses by the predicted quadratic model were found in consensus with the obtained values. Our results well depicted the favourable conditions to further scale-up the volumetric yield of other relevant recombinant enzymes and proteins.

## Introduction

Cellulases with distinctive and manifold applications in a diverse array of fields, hold the third highest position in the global commercial enzymes market^1^. The demand of cellulases has been escalating day by day in a way that, to cope up with its mandate is a tangible challenge in recent years. High efficacy of recombinant microbes also results in higher productivity and expression of recombinant enzymes, ensuring their availability and utilization at an extreme level^2^. The production enzymes with desired characteristics is imperative to encounter the limitations of naturally produced enzymes^3^. Optimum bacterial growth, superlative production and top-notch expression of recombinant enzymes are significantly influenced by various fermentation parameters. Nature of production host organism and selection of plasmid vector is crucial to the expression of a particular enzyme^4^. Genetic manipulation for the production of heterologous proteins in *E. coli* has been progressively improved. Achieving a fast growth rate up to one generation per twenty minutes, under optimized conditions makes it an attractive procedure^5,6^. As compared to other hosts, competent cells preparation is relatively less complicated and transformation of foreign plasmid DNA in *E. coli* is eminently successful and efficient^7,8^. High yield of heterologous proteins can be achieved by amalgamation of simple and complex media components ranging from simple carbohydrates to complex peptones, tryptones, yeast extract and so forth. Optimization of media composition aids in attaining high productivity with cost reduction. Similarly, the selection of a media-microbe match can also enhance the yield^9,10^. A strategical approach for the induction of recombinant cellulase included the supplementation of attributed, economical and non-toxic inducers. Lactose fits best as an inducer according to the above mentioned properties^11^. Lactose being metabolized as a carbon source by the cells for optimal growth, although results in maximum biomass but ultimately becomes inadequate for the expression of recombinant proteins, consequently costs at prolonged induction time. Reinforcing the lactose supplementation with glycerol or glucose may relieves the protracted induction time. However, lactose and IPTG have their own merits and demerits as inducers^12,13^. Recombinant production as well as activity of enzymes diminutions when fluctuating from the optimum temperature and pH. Bacteria have an ability to survive in an array of environmental conditions including extreme temperatures, acidic or alkaline pH levels^14^.

Aerobic fermentation processes demand provision of oxygen at the rate to sufficiently satisfy the growth need of organism. Aeration is the most important and critical factor for the efficiency of fermentation process and is achieved by supplying the bacterial culture with adequate efflux of oxygen to the fermentation broth^15^. Proper aeration leads to saturation of dissolved oxygen in bacterial cell culture, resulting in an enhanced bacterial growth and production of cellulase up to many folds in comparison to non-agitated or static cultures^16^. Selection of *E. coli* preferred codon and introduction of single codon silent mutation in the primer sequence aids to achieve lower structural stability leading towards higher expression^17^. On the basis of above background, current study intended to achieve overproduction and overexpression of recombinant cellulase by employing certain molecular engineering techniques and optimization of fermentation parameters by response surface methodology. Optimization of growth media, supplementing with enriched nutrients, and fermentation crucial parameters will help to develop cost-effective media to achieve higher productivity with reduced induction time for the industrial benefits.

## Results

### Fermentation conditions optimization using response surface methodology (RSM)

Significant fermentation parameters including fermentation temperature, pH and agitation rate were optimized by varying the levels of these factors using central composite design for achieving higher cell growth with maximum CMCase activity. Table 2 depicted the obtained values of the responses i.e. cell growth and CMCase activity. Statistical testing of the model using analysis of variance (ANOVA) is presented in the Table 3A and B for cell growth and CMCase activity, respectively. The *F*-values of the response surface quadratic model were evaluated to be 56.56 and 30.19 for cell growth and CMCase activity, respectively, which implies that the model is significant. Fit statistical values well augmented the ANOVA. The determination coefficient value (*R*^2^) was estimated to be 0.9826 and 0.9679, for growth and activity, respectively, that in accordance with the insignificant lack of fit (*F*-value 0.25) depicted that the predicted quadratic model was able to explain 99.23% of the results. The predicted determination coefficient (*R*^2^) of 0.8287 and 0.9002 was in rational agreement with the adjusted *R*^2^ of 0.9653 and 0.9359, for growth and activity respectively, i.e. acquiring the difference of less than 0.2, indicated that the predicted model was unable to explain merely approximately 1% of the variations. These estimations confirmed that the predicted quadratic models were significant (*p* < 0.0001) adequately suitable for predicting both the responses i.e. cell growth and CMCase activity as well as the relationship between the response and the independent variables. Moreover, the quadratic factors (*A*^2^, *B*^2^ and *C*^2^) showed significance at* p* < 0.0001.

**Figure 1:**
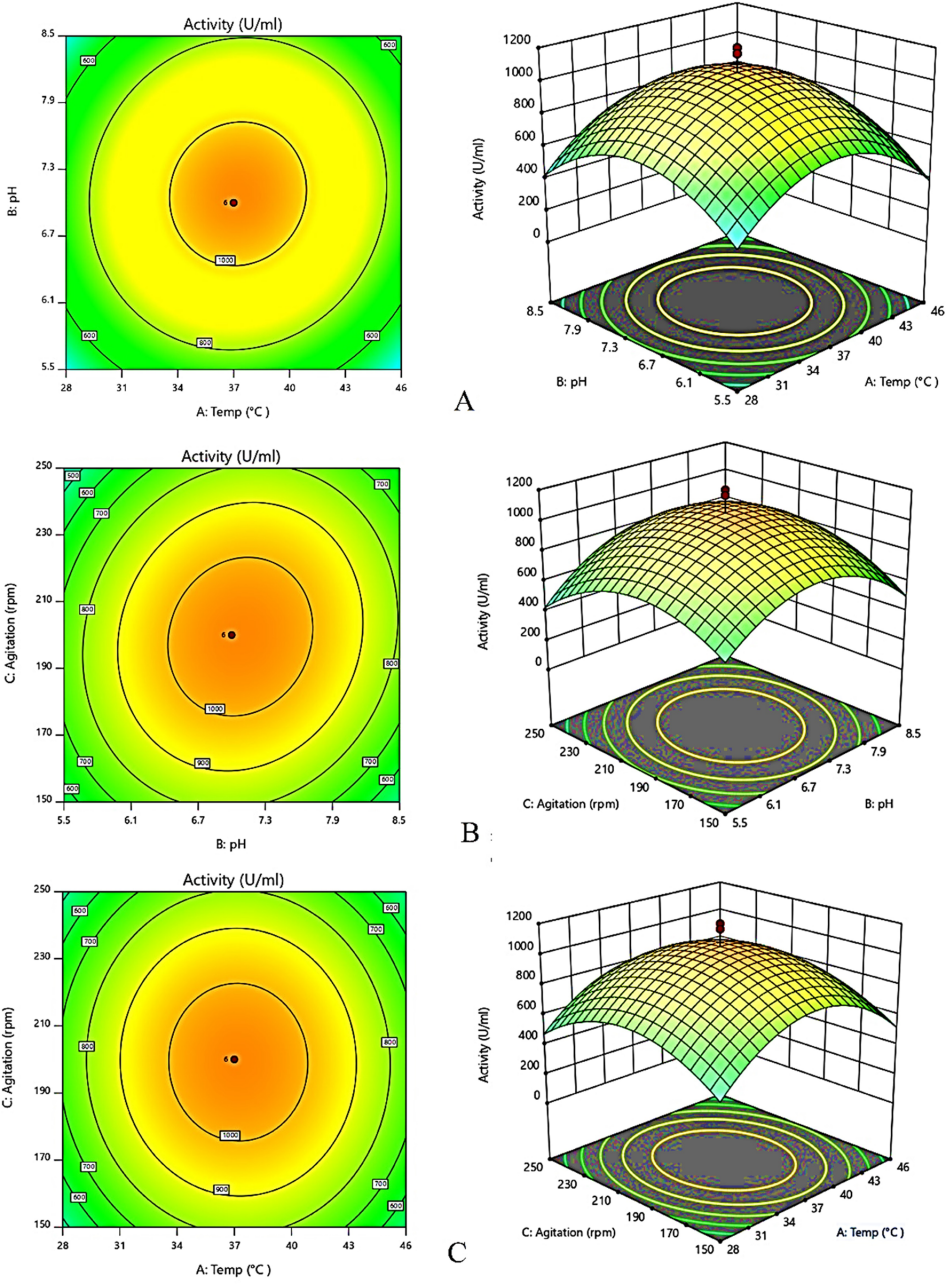
2D contour plots and 3D response surface plot for recombinant cellulase production depicting interactive effects between (**A**) temperature and pH, (**B**) pH and agitation rate and (**C**) temperature and agitation rate. Design expert version 11 software used from web site: www.statease.com.

To investigate the relationship between dependent and independent variables and for determination of their optimum levels, 2D contour plots as well as 3D response surface plot were generated by plotting one of the responses against any two independent variables whereas the third variable was maintained at its middle (0) levels. Figure 1(A) representing the interaction effect between fermentation temperature and pH on CMCase activity at constant agitation rate (200 rpm) revealed that higher activity was achieved at temperature higher than 35 °C but lowered than 45 °C corresponding to the neutral pH. At pH towards basic environment with the lower temperature caused negative effect on the activity. Figure 1(B) showing the probable interactive effect of pH and agitation rate at constant temperature (37 °C) on the activity depicted that increasing agitation rate above 200 rpm increasing or decreasing pH. This fluctuations of pH does not show any significant increase in the activity rather acidic and basic fermentation environment leads to decline in the activity irrespective of agitation rate. Figure 1(C) displaying the effect on temperature and agitation rate showed insignificant relation on the activity as lowered and extremely high temperatures caused decline in the activity irrespective of increase in the agitation rate.

### PCR amplification and cloning in expression vector

Various combinations of silent mutations at initial codons were examined to minimize the translation-hindering stable secondary structures at 5′ of mRNA that has been reported to negatively affect the protein expression. *ΔG* values were estimated from RBS (ribosomal binding site) of mRNA to + 10 codon. Out of all possible combinations examined (Table 1), silent mutation at second codon (GTA > GTT) with *ΔG* − 2.80 was found to have promising influence on the translation as compared to the native sequence having *ΔG* − 4.0. Purified plasmid containing recombinant cellulase gene (*celA-BC*) was amplified through polymerase chain reaction and PCR product analysed by 1% agarose gel electrophoresis, showed successful amplification with respect to the expected size i.e. 1.6 kb. The amplified product was purified followed by the double restriction and ligation of the restricted gene and pET22b(+). The analysis of the clones verified the successful cloning into the expression vector, pET22b(+) and formation of recombinant vector of 7.1 kb.

**Table 1:** Sequences and their respective *ΔG* values (RBS: ribosome binding site of pET22b vector).

Sequence (RBS > + 10 codon)	*ΔG* (kcal/mole)
**Native**	− 4.0
AAGGAGATATACAT ATG GTA TCA GGC AAT TTG AAG GTT GAA TTC	
RBS + 1 + 2 + 3 + 4 + 5 + 6 + 7 + 8 + 9 + 10	
AAGGAGATATACAT ATG GTT TCA GGC AAT TTG AAG GTT GAA TTC	− 2.8
AAGGAGATATACAT ATG GTG TCA GGC AAT TTG AAG GTT GAA TTC	− 4.0
AAGGAGATATACAT ATG GTC TCA GGC AAT TTG AAG GTT GAA TTC	− 5.4
AAGGAGATATACAT ATG GTA TCT GGC AAT TTG AAG GTT GAA TTC	− 4.8
AAGGAGATATACAT ATG GTA TCG GGC AAT TTG AAG GTT GAA TTC	− 4.0
AAGGAGATATACAT ATG GTA TCC GGC AAT TTG AAG GTT GAA TTC	− 4.3
AAGGAGATATACAT ATG GTA TCA GGT AAT TTG AAG GTT GAA TTC	− 2.8
AAGGAGATATACAT ATG GTA TCA GGA AAT TTG AAG GTT GAA TTC	− 2.8
AAGGAGATATACAT ATG GTA TCA GGG AAT TTG AAG GTT GAA TTC	− 4.8
AAGGAGATATACAT ATG GTA TCA GGC AAC TTG AAG GTT GAA TTC	− 6.4
AAGGAGATATACAT ATG GTA TCA GGC AAT TTA AAG GTT GAA TTC	− 4.0
AAGGAGATATACAT ATG GTA TCA GGC AAT CTG AAG GTT GAA TTC	− 6.1
AAGGAGATATACAT ATG GTA TCA GGC AAT TTG AAA GTT GAA TTC	− 3.8
AAGGAGATATACAT ATG GTA TCA GGC AAT TTG AAG GTA GAA TTC	− 2.8
AAGGAGATATACAT ATG GTA TCA GGC AAT TTG AAG GTG GAA TTC	− 2.8
AAGGAGATATACAT ATG GTA TCA GGC AAT TTG AAG GTC GAA TTC	− 4.8
AAGGAGATATACAT ATG GTA TCA GGC AAT TTG AAG GTT GAG TTC	− 4.0
AAGGAGATATACAT ATG GTA TCA GGC AAT TTG AAG GTT GAA TTT	− 4.0

### Validation of RSM through shake flask fermentation

**Figure 2:**
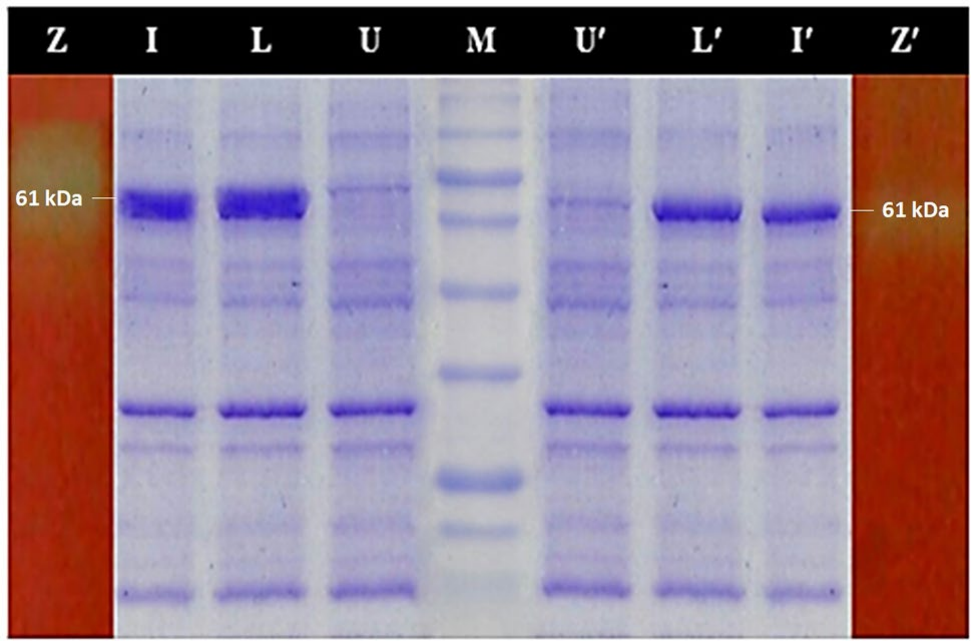
SDS-PAGE expression analysis for recombinant cellulase. M: Protein marker (BioLabs-P7706L), Lane U, U′: Un-induced samples, Lane I, I′: Samples with IPTG induction, Lane L, L′: Lactose induction, Lane Z, Z′: Zymogram of recombinant cellulase against CMC; in modified M9NG and LB media respectively.

To augment the bacterial growth and production of recombinant cellulase, numerous fermentation parameters including culture medium, temperature, pH and agitation rate were optimized during shake flask fermentation. Recombinant *E. coli* was best fermented in modified M9NG media when auto-induced with 10 mM lactose overnight that produced biomass concentration of 6.9 gl^−1^ with 30% expression of recombinant cellulase. Bacterial growth obtained from IPTG induction in modified M9NG medium was 2.5 gl^−1^ with 30% expression and was plateaued after 4 h of induction. Therefore, it indicated that both the inducers in modified M9NG media are proficient for aggrandized production of recombinant cellulase. However, bacterial growth obtained in LB media was much lowered (3.5 gl^−1^) that resulted in least expression i.e. 18%. Expression analysis with 12% SDS-PAGE confirmed the expression of all the induced samples at the band corresponding to their respective molecular weight (61 kDa) against un-induced sample as a negative control. Confirmation of expressed protein as cellulase was also done by zymography by using CMC as substrate. Zymogram also presented recombinant cellulase expressed at its corresponding molecular weight i.e. 61 kDa (Fig. 2). Moreover, activity of recombinant cellulase against CMC was estimated to be 1185 U/mg which was in reasonable agreement with the predicted activity by the model.

### Apposite culture media for enhanced recombinant cellulase production

**Figure 3:**
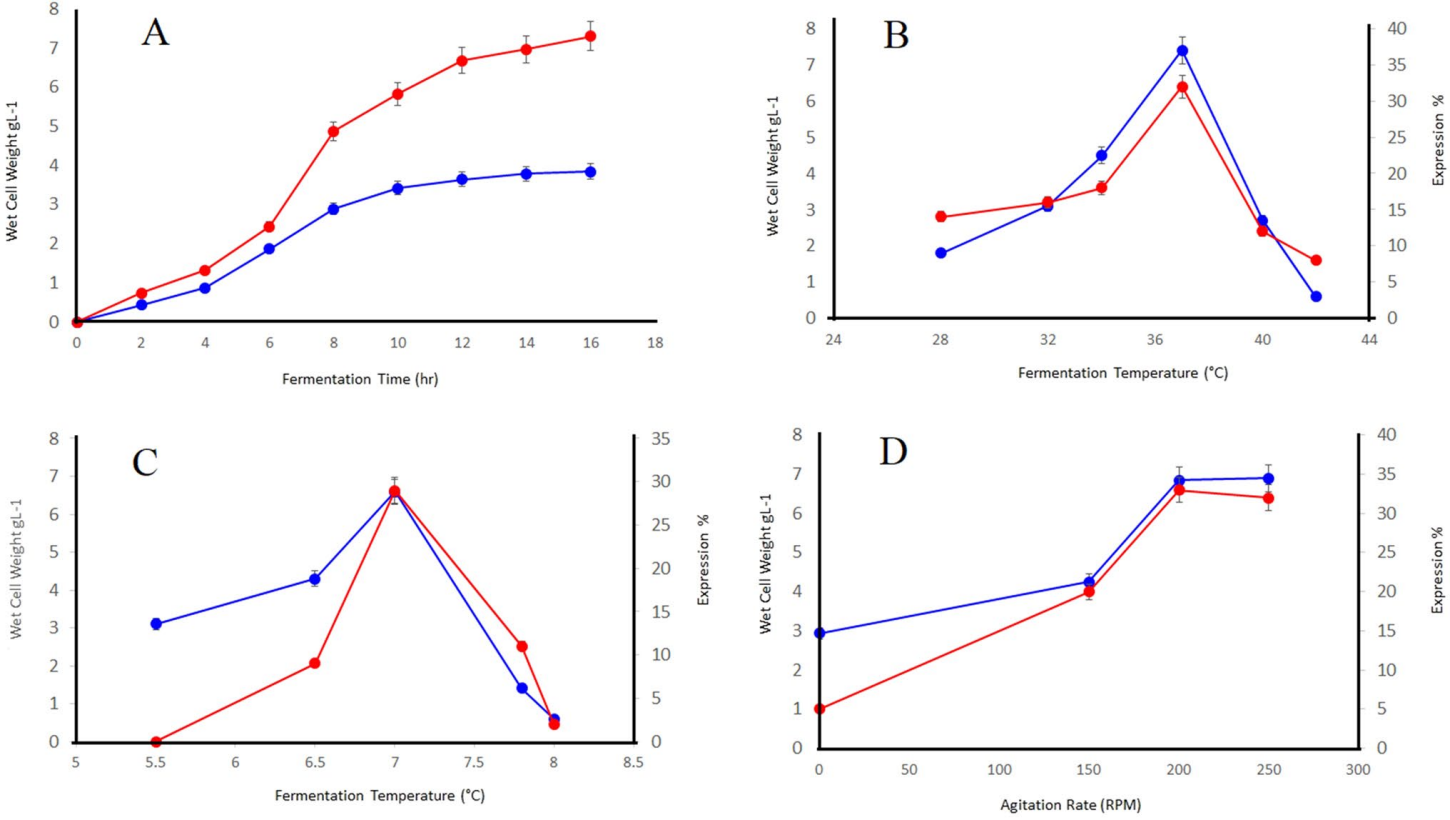
(**A**) Time course of recombinant cellulase fermentation by *E. coli* BL21 (DE3) in LB (□) and modified M9NG media (♦) over 16 h of shake flask fermentation. Effect of (**B**) temperature, (**C**) pH and (**D**) agitation rate on recombinant cellulase fermentation by *E. coli* BL21 (DE3) determined by incubating at different fermentation temperature, pH and agitation rates respectively. Wet cell weight (♦), recombinant cellulase expression (□).

Recombinant *E. coli* fermented in modified M9NG media yielded the higher biomass concentration of 6.9 gl^−1^ with 30% expression of recombinant cellulase (Fig. 3A). However, the enzyme was not pronouncedly expressed in LB media over the 16 h of fermentation. Lowered production and expression in LB media specified that solely complex media components viz. tryptone and yeast extract are not sufficient for the higher production and expression of recombinant cellulase in short time period. Modified M9NG media supplemented with 3 mM glucose and 24 mM glycerol along with resting salts and nutrients resulted in better production and expression in possible least induction time.

### Optimum temperature for enhanced recombinant cellulase production

A trend of optimal growth at mesophilic temperature was observed for recombinant cellulase during the present study. Greater expression (30%) corresponding to higher biomass (6.9 gl^−1^) was achieved when incubated at 37 °C (Fig. 3B). At low temperature, the cellulase expression was minimal due to less microbial growth and the cells underwent death phase at the high temperature.

### Optimum pH for enhanced recombinant cellulase production

Highest biomass concentration (6.8 gl^−1^) was yielded when fermentation run was carried out in pH 7, that superlatively expressed recombinant cellulase with 29% expression. Beyond this limit in either way, the recombinant cellulase gradually lost the expression however, acidic pH adversely affected the fermentation resulted in null expression (Fig. 3C).

### Optimum agitation rate for enhanced recombinant cellulase production in E. coli

Limiting oxygen supply caused by inadequate stirring is the major bottleneck in high cell density fermentation run. Higher yield (6.9 gl^−1^) was obtained from the culture of *E. coli* BL21 (DE3) when fermented with moderate agitation rate i.e. 200 rpm sufficient for the surface aeration, dissolved oxygen concentration and oxygen supply to the site of oxidative phosphorylation in the cell. Increasing agitation speed did not exhibited pronounced effect on the production (Fig. 3D).

## Discussion

*Escherichia coli* is a widely used advantageous host cell for the heterologous gene^18,19^. Several factors have been reported to control the bacterial growth conditions and yield of recombinant proteins. These factors include the concentration, pH, temperature of medium, and composition of various salts/additives, plasmid number, IPTG concentration. *E. coli* has an ability to show growth in culture even at lag phase of growth^20,21^. We have previously reported the cloning, purification and characterization of endoglucanase from *Clostridium thermocellum*^22^. The present study describes our experiments for the scaling-up of fermentation conditions to improve the yield and quality of endoglucanase by exploiting various influencing factors.

Response surface methodology (RSM), is a bunch of multivariant statistical, and mathematical tools for optimization of the culture conditions and bacterial response^23^. In the first step, the effect of agitation (rpm), temperature and pH of culture was determined. Maximum bacterial biomass, and CMCase activity was found at pH 7, 37 °C and 200 rpm (Table 2). The quadratic model for cell growth optimized in the present study has shown significant improvement in the bacterial biomass and enzyme activity (Table 3A, B). Large *F*-values with corresponding small *p* values (i.e. < 0.0001) for both the responses highly signifies the respected coefficients as generally depicted^24^. The effect of independent variables has been described by 2D contour plots as well as 3D response surface plot. In the literature, these plots have been applied to provide a clear idea about the impact of variables^25,26^. It indicates that a temperature above 35 °C but lowered than 45 °C has positive effect on enzyme activity at neutral pH (Fig. 1A–C). Similar findings have been reported in the literature^22^. Different silent mutations were examined at initial codons to minimize the reported translation-hindering stable secondary structures at 5′ of mRNA^27^. The binding affinities (*ΔG values*) of native and mutated codons were determined by Mfold web server^28,29^. The mutation GTA > GTT has shown a promoting impact on mRNA binding with the ribosomes^30^. The best yield of active enzyme was obtained in modified M9NG media under 10 mM lactose. It has given 6.9 gl^−1^ biomass and about 30% expression level. Similar expression level was obtained by 0.5 mM IPTG, however the final biomass was restricted to 2.5 gl^−1^. On the other hand LB media could generate 3.5 gl^−1^ biomass with only 18% expression. The expressed enzyme gave a band at 61 kDa on SDS-PAGE (Fig. 2). Lactose based induction has been already reported for its merits in recombinant protein synthesis^31^. It can also promote the soluble fraction of proteins. Recently, the strains with high lactose intake abilities have been introduced in for the optimization of experiments with better yield^32,33^. The enzyme activity with CMC was found 1185 U/mL which is similar to the model predicted activity. In our studies, the enriched M9NG media with 3 mM Glucose and 24 mM glycerol gave highest yield in the shortest time (Fig. 3A). Glucose and glycerol are in limelight for researchers desiring to achieve higher volumetric yield with reduced induction time for a wide range of bacterial recombinant proteins^34^. The optimum enzyme yield was obtained at mesophilic growth temperatures (Fig. 3B). According to the literature, for the production of various recombinant enzymes, *E. coli* BL21 (DE3) promotes higher yield and expression when fermented at around 37 °C of incubation temperature^35,36^. Moreover, higher temperatures corresponded to the lowered growth with least expression predominantly due the reduced glucose uptake rate and lowered saturated oxygen concentration^37^. Owing to the extreme stress and metabolic burden imposed onto the bacterial cells by the induction and growth at higher temperatures, fermentation resulted in decreased production and expression^38^. Moreover, deficient oxygen solubility at higher temperature may responsible for the alteration of TCA-cycle’s gene expression ultimately resulted in heavily accumulation of organic acid viz. acetic acid consequently occasioned in lowest yield and expression^39,40^. Optimum bacterial biomass and endoglucanase activity was found at neutral pH. Most probably, owing to the neutrophilic nature of *E. coli*, neutral pH was found to be quintessential environment for the excessive bacterial growth with top-notch expression i.e. likewise reckoned by previous researches^41,42^. Fluctuation of proton electrochemical gradient or imbalanced extra-intracellular pH that foist huge stress on the cells and affects growth and production can be considered as main factor^43^. Acidic pH corresponded to the least cell growth with negligible expression primarily due the cytoplasmic pH acidification below the threshold level and consequent hindrance to the cellular function^44^. Therefore, lowered pH of the medium attributed to the lowered growth rate of *E. coli* subsequently resulted in lowered growth and production of recombinant cellulase. The agitation rate of 200 rpm gave maximum yield, there was negative effect of further increase in agitation. On the other hand, static fermentation accountable for oxygen downshift resulted in lowered biomass and production. Owing to the inequity in rapid glucose uptake and restricted oxygen solubility in the cell culture due to the high viscosity of the culture medium^43^, a representative conflict between raised oxygen demand by the facultative cells at exponential phase and low level of dissolved oxygen may ascribed to the metabolism overflow and subsequent high glycolytic flux that adversely affected the bacterial growth and enzymatic production. Agitation greater than moderate rate is not recommended due to the possible sheering effects on the cells^45^. The present study signifies the predicted quadratic model using central composite design for achieving overproduction with maximum activity of recombinant cellulase. The study enlightened the importance of numerous fermentation parameters and their influence on the bacterial growth and activity. The interaction effect of temperature and pH on the CMCase activity was found to be highly significant. The recombinant cellulase was superlatively fermented in modified M9NG media when incubated at 37 °C, pH 7.0 with the agitation rate of 200 rpm.

**Table 2:** Quadratic model predicted responses of the fermentation parameters by using central composite design.

Run	Space type	Temperature (°C)	pH	Agitation (rpm)	Cell growth (g/l)	CMCase activity (U/ml)
1	Center	(0)37	(0)7	(0)200	6.5	1165
2	Center	(0)37	(0)7	(0)200	6.5	1165
3	Factorial	(− 1)28	(− 1)5.5	(+ 1)250	2.2	130
4	Factorial	(+ 1)46	(+ 1)8.5	(− 1)150	1.5	325
5	Center	(0)37	(0)7	(0)200	6.5	1165
6	Factorial	(− 1)28	(+ 1)8.5	(− 1)150	1.8	140
7	Factorial	(+ 1)46	(− 1)5.5	(− 1)150	2.5	280
8	Center	(0)37	(0)7	(0)200	6.5	1165
9	Factorial	(− 1)28	(− 1)5.5	(− 1)150	2.8	320
10	Factorial	(+ 1)46	(+ 1)8.5	(+ 1)250	2.5	330
11	Factorial	(− 1)28	(+ 1)8.5	(+ 1)250	3.5	270
12	Factorial	(+ 1)46	(− 1)5.5	(+ 1)250	2.5	155
13	Axial	(0)37	(+ α)9.52269	(0)200	3.5	250
14	Center	(0)37	(0)7	(0)200	6.5	1165
15	Axial	(0)37	(0)7	(− α)115.91	3	300
16	Center	(0)37	(0)7	(0)200	6.5	1165
17	Axial	(+ α)52.1361	(0)7	(0)200	0.65	80
18	Axial	(0)37	(0)7	(+ α)284.09	3.5	345
19	Axial	(0)37	(− α)4.47731	(0)200	2	110
20	Axial	(− α)21.8639	(0)7	(0)200	0.7	95

**Table 3:** Analysis of variance (ANOVA) of quadratic model for cell growth of *E. coli* BL21 (3A) and CMCase activity (3B).

Source	Sum of squares (SS)	Degree of freedom (df)	Mean square (MS)	F-value	*p* value	
**(3A)**						
Model	81.84	9	9.09	56.56	< 0.0001	Significant
A-temp	0.1403	1	0.1403	0.8725	0.3746	
B-pH	0.2433	1	0.2433	1.51	0.2498	
C-agitation	0.6333	1	0.6333	3.94	0.0785	
AB	0.2112	1	0.2112	1.31	0.2812	
AC	0.0012	1	0.0012	0.0078	0.9317	
BC	1.36	1	1.36	8.47	0.0173	
A^2^	55.33	1	55.33	344.15	< 0.0001	
B^2^	21.66	1	21.66	134.75	< 0.0001	
C^2^	15.87	1	15.87	98.71	< 0.0001	
Residual	1.45	9	0.1608			
Lack of fit	1.45	5	0.2894			
**(3B)**						
Model	3.028E+06	9	3.365E+05	30.19	< 0.0001	Significant
A-temp	3070.40	1	3070.40	0.2755	0.6123	
B-pH	12,638.31	1	12,638.31	1.13	0.3147	
C-agitation	796.85	1	796.85	0.0715	0.7952	
AB	8450.00	1	8450.00	0.7582	0.4065	
AC	450.00	1	450.00	0.0404	0.8452	
BC	25,312.50	1	25,312.50	2.27	0.1661	
A^2^	1.507E+06	1	1.507E+06	135.19	< 0.0001	
B^2^	1.217E+06	1	1.217E+06	109.23	< 0.0001	
C^2^	8.321E+05	1	8.321E+05	74.66	< 0.0001	
Residual	1.003E+05	9	11,144.79			
Lack of fit	23,928.11	5	4785.62	0.2506	0.9193	Insignificant

## Conclusions

The RSM model developed in the present study along with the selected media and additives provides better yield and activity of endoglucanase. Optimization of these fermentation parameters will help to enhance the volumetric production of recombinant cellulase in cost-effective media in least time during batch fermentation.

## Methods

### Predicted model for fermentation conditions optimization using response surface methodology (RSM)

Response surface methodology was designed to achieve highest culture density of *E. coli* BL21 (DE3) with maximum recombinant cellulase activity using a set of fermentation conditions. Various factors including fermentation temperature (°C), pH and agitation rate (rpm) were investigated systemically using central composite design (CCD) (Design Expert software, v11, Stat-Ease Inc, 11.1.2.0), known to be suited well to fit a quadratic surface for process optimization. Each numeric factor (temperature, pH and agitation rate) was set to five levels i.e. + α and − α (axial points), + 1 and − 1 (factorial points) and 0 (center point). The whole design comprised of 20 experimental runs including six replicates at the center of the design to estimate the pure error. The experiments were run in triplicate at each design points conducted in randomized order. Table 2 enlisted all the chosen ranges and levels of all the three factors whereby 0 (center point value) represented the frequently reported factor level in the literature. Moreover, low and high values of the factors were determined in accordance with the center point that remained as a middle value of the low and high ranges. Responses of all sets of experimental runs with respect to optimized design and conditions were estimated in terms of cell growth and CMCase activity using the Design Expert software. Analysis of variance (ANOVA) was examined for the statistical testing of the predicted model. *F-*value and *p*-value were analyzed to check the significance of the quadratic model.

### Validation of the predicted quadratic model

For verification of the predicted model, successful recombinant cellulase production was carried out in *E. coli* BL21 (DE3) and overproduction with overexpression was achieved in modified M9NG media by employing the optimized conditions.

### Bacterial strain and vector

Anaerobic cellulolytic, *Clostridium thermocellum* ATCC 27405 was used for endoglucanase gene having catalytic domain of celA (Genebank Ac. No. K03088) at C-terminus of a family 3a carbohydrate binding module from the scaffolding protein, cipA (Genbank Ac. No. L08665). *Escherichia coli* BL21 (DE3)—CodonPlus RIPL strain [E. coli B F– ompT hsdS (rB– mB–) dcm + Tetr gal λ (DE3) endA Hte (argU ileY proL leuW Camr)] from Novagen (Madison, Wis.) and pET22b (+) as an expression vector were used for the expression of recombinant cellulases.

### PCR amplification and product analysis

PCR amplification was carried out by using the primers as described by Sajjad et al., 2012. However, silent mutation was introduced at the second codon (GTA → GTT) of the native sequence of the primer used for the cloning, previously^12^. The native primer having nucleotide sequence 5′ cacatatg**gta**tcaggcaatttgaagg 3′ (ΔG − 4.0 kcal/mol) was replaced by a primer with above mentioned mutation i.e. 5′ cacatatg**gtt**tcaggcaatttgaagg 3′ (ΔG − 2.8 kcal/mol). PCR amplification conducted over 30 cycles underwent denaturation at 94 °C for 45 s, annealing at 58 °C for 45 s and extension at 72 °C for 90 s. Final extension was executed for 20 min at 72 °C. PCR product was checked on 1% agarose gel and DNA purification or gene cleaning was done with the help of MONARCH DNA Gel Extraction Kit (NEB #T1020) by following manufacturer’s instructions.

### Cloning in expression vector pET22b (+)

Purified plasmid containing cellulase gene and pET22b (+) were restricted with *Sac*I and *Nde*I restriction enzymes. Restriction of the plasmid was checked with the help of 1% agarose gel electrophoresis and desired DNA fragments were purified using MONARCH DNA Gel Extraction Kit Protocol (NEB #T1020) using the manufacturer’s guide. Purified restricted cellulase was then ligated into linearized pET22b (+). The ligation mixture was incubated on ice for 1 h and then at 20 °C for overnight. Competent cells of *E. coli* BL21 (DE3) were then transformed with this ligation mixture containing desired heterologous plasmid by conventional heat shock method. For the screening of transformants, the transformed cells were spread over the LB-agar plates (1.5% agar containing 50 μg ml^−1^ ampicillin) and was incubated at 37℃ overnight. A single colony was isolated and inoculated in 10 ml of LB medium and 20 ml modified M9NG media, both containing 100 µg ml^−1^ ampicillin. Both the flasks were incubated at 37 °C, 200 rpm on shaking incubator to grow overnight.

### Recombinant cellulase production

Recombinant cellulase production was examined in LB and modified M9NG media in shake flask fermentation. Components of modified M9NG media included 1% tryptone, 0.5% yeast extract and 0.5% NaCl, along with resting salts and components i.e. 25 mM K_2_HPO_4_, 25 mM NaH_2_PO_4_, 15 mM NH_4_Cl, 8 mM MgSO_4_.7H_2_O, 1 mM Trace metals; each (CuSO_4_, MnCl_2_, CaCl_2_, MoO, FeCl_3_), 24 mM Glycerol, 3 mM Glucose and 10 mM Lactose. Two sets of 20 ml each of LB and modified M9NG media were prepared and inoculated with 20 µl of their respective overnight cultures, both supplemented with 100 μg ml^−1^ ampicillin and incubated at 37℃, 200 rpm on incubator shaker till OD_600nm_ of 0.6–0.7 was attained. Expression of recombinant cellulase was induced, separately with 10 mM lactose and 0.5 mM IPTG and again incubated at 37℃, 200 rpm on incubator shaker for further 2–6 h. Samples were drawn after every 2 h of fermentation. In a parallel experiment, cells expressing recombinant cellulase were also auto-induced with 10 mM lactose in 20 ml of LB and modified M9NG media containing with 100 μg ml^−1^ ampicillin. The cultures were grown overnight (12–15 h of induction) at 37℃, 200 rpm. Samples were drawn after 4 h and 15 h (minimum and adequately maximum hours for auto-induction, respectively). After fermentation, the induced cells were centrifuged at 6500 × *g* for 10 min to be harvested. Supernatant was decanted off and air-dried cell pellets were resuspended in 20 mM Tris buffer; pH 7.0 to a final OD_600nm_ of 10 and samples were prepared for conducting expression analysis and CMCase activity assay.

#### Optimization of fermentation conditions

Different fermentation parameters were optimized during the shake flask fermentation so as to operate fermentation at large scale with these optimized conditions in order to evade unsuccessful batches, complexities and time wastage. For determination of suitable and appropriate nourished growth media, recombinant cellulase containing culture was fermented in LB and modified M9NG to enhance the bacterial growth and production of enzyme. The culture was fermented at 28 °C, 32 °C, 37 °C, 40 °C and 42 °C to determine the optimum temperature. The pH was optimized over a broad range of pH ranging from 5.0 to 9.0. Optimum agitated rate for the optimum bacterial growth was also determined by fermenting the culture statically and agitated at 150, 200 and 250 rpm. Moreover, culture was auto-induced as well as induced on the basis of time/OD_600nm_ over the period of time ranging from 4 to 16 h.

### Expression analysis and CMCase activity assay for recombinant cellulase

Expression of recombinant cellulase in terms of total cell protein was analyzed on 12% SDS-polyacrylamide gel, stained with coomassie brilliant blue R-250 (Sigma Aldrich, #27816), by the standard electrophoresis protocol. Confirmation of the expression of recombinant cellulase was done by the zymographic analysis on CMC-agar plate. Zymography and CMCase activity assay was done according to the modified protocol as described by Sajjad et al., 2012.

## Data Availability

All the relevant data is available with the corresponding author.

## Abbreviations

RSM: Response surface methodology
CCD: Central composite design
RPM: Revolution per minute
LB: Luria-Bertani
RBS: Ribosome binding site
SDS-PAGE: Sodium dodecyl sulphate
CMC: Carboxy methyl sephadex
IPTG: Isopropyl β-d-1-thiogalactopyranoside
